# The barley disease resistance protein *AvrPphB Response 1* exhibits nucleocytoplasmic localization

**DOI:** 10.17912/micropub.biology.002041

**Published:** 2026-05-19

**Authors:** Matthew Helm, Ariana Myers, Rachel Kelly, Namrata Jaiswal, Anjali Iyer-Pascuzzi, Steven Scofield

**Affiliations:** 1 Crop Production and Pest Control Research Unit, U.S. Department of Agriculture, Agricultural Research Service (USDA-ARS), West Lafayette, IN, USA; 2 Department of Botany and Plant Pathology, Purdue University, West Lafayette, IN, USA

## Abstract

Plant immunity is partly mediated by intracellular nucleotide-binding leucine-rich repeat (NLR) proteins that detect pathogen effector activity, with NLR function often linked to subcellular localization. We previously showed the barley NLR *AvrPphB Response 1* (PBR1) recognizes the *Pseudomonas syringae* effector protease AvrPphB; however, PBR1 localization and the factors influencing it were unknown. Using confocal microscopy following transient expression in *Nicotiana benthamiana* , we examined full-length PBR1, individual domains, truncations, an N-terminally tagged variant, and a signaling-inactive mutant. PBR1 and all variants localized predominantly to the nucleocytosol, demonstrating that PBR1 localization is independent of domain composition, activation state, or N-terminal tag placement.

**Figure 1. The barley NLR immune receptor PBR1 localizes to the nucleocytosol independent of individual domains, truncations, fluorescent tag orientation, or immune activation state.** The indicated super Yellow Fluorescent Protein (sYFP)-tagged constructs were transiently expressed in *N. benthamiana* using agroinfiltration, and leaf sections imaged using laser scanning confocal microscopy. Confocal micrographs are of single optical sections. Free sYFP (3xHA:sYFP) was used as a reference control for nucleocytoplasmic localization (Helm et al., 2022; Jaiswal et al., 2023). White arrowheads in panel A indicate sYFP fluorescence signal detected in the nucleus and cytoplasm. The scale bars shown represent 20μm (panels A, B, C, E, F, H, and I) and 50μm (panels D and G). Two independent experiments were performed with similar results. The findings from one experiment are shown.

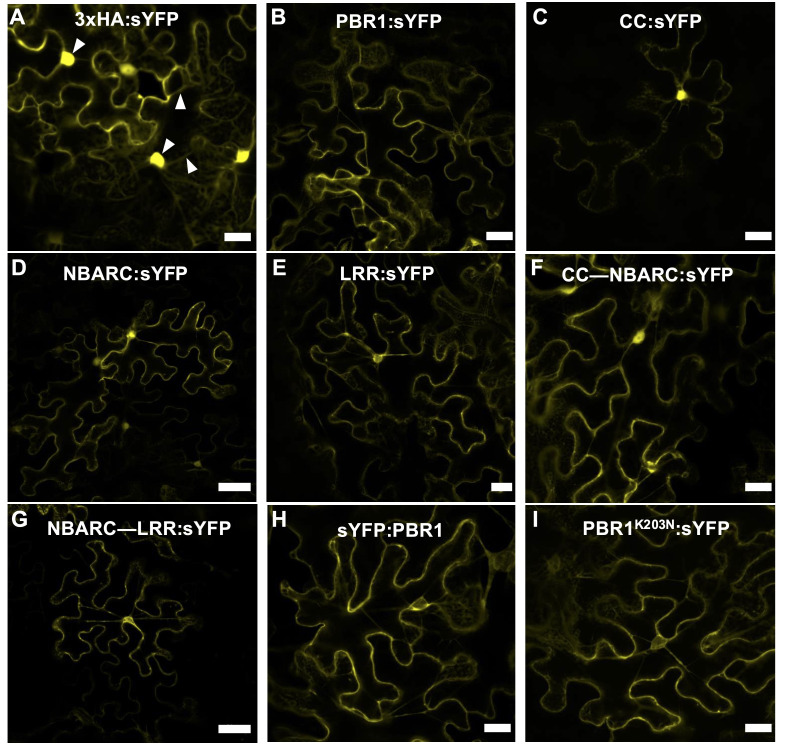



## Description

Plant immunity relies, in part, on intracellular receptors known as nucleotide-binding leucine-rich repeat (NLR) proteins whose primary function is to detect the activity of pathogen-derived effector proteins (Jones and Dangl, 2006). Upon effector recognition, plant NLR proteins trigger a rapid localized cell death response, termed the hypersensitive response, which is often linked to limiting pathogen growth and systemic spread (Coll et al., 2011; Jones and Dangl, 2006; Klement and Goodman, 1967; Kourelis and van der Hoorn, 2018; Ngou et al., 2021, 2022; van der Burgh and Joosten, 2019). Most characterized NLR proteins share a conserved three-part organization, comprising a variable N-terminal signaling domain, a central NB-ARC domain, and a C-terminal leucine-rich repeat (LRR) domain (Caplan et al., 2008; McHale et al., 2006; Takken and Tameling, 2009). Variation within the N-terminal domain distinguishes two major classes of plant NLR immune receptors, defined by the presence of either a coiled-coil (CC) or Toll/interleukin-1 receptor-like (TIR) domain (McHale et al., 2006; Meyers et al., 2003). Functionally, the CC domain of many plant NLR proteins serves as a key signaling module that often promotes activation of the hypersensitive reaction, although full immune activation for some NLR immune receptors requires coordinated interactions among multiple domains (Bai et al., 2012; Baudin et al., 2017; Cesari et al., 2016; Collier et al., 2011; Hamel et al., 2016; Jaiswal et al., 2023; Kim et al., 2018; Wang et al., 2015; Wang et al., 2019a, b). The NB-ARC region plays a central regulatory role by coordinating nucleotide binding, intramolecular interactions, and oligomerization, and contains conserved motifs critical for NLR activation, such as the Walker A motif (Collier and Moffett, 2009; Maekawa et al., 2011; Takken et al., 2006; Wang et al., 2019a, b). The LRR domain is the most variable region and often contributes to effector recognition, thereby enabling conformational changes required for NLR activation (Ellis et al., 2000; Krasileva et al., 2010; Wang et al., 2019a, b).

Plant NLR proteins also exhibit diverse subcellular localizations, which often correspond to the sites where they detect their cognate effectors (Qi and Innes, 2013). For instance, the *Arabidopsis thaliana* NLR immune receptor ZAR1 assembles a resistosome at the plasma membrane following activation, where it functions as a calcium-permeable channel to initiate immune signaling (Bi et al., 2021; Hu et al., 2020; Wang et al., 2019a; Wang et al., 2019b). Similarly, the plasma membrane-associated NLR from *Arabidopsis thaliana* , RPM1, monitors effector-induced perturbations of host proteins at the cell periphery (El Kasmi et al., 2017; Gao et al., 2011). For some NLR immune receptors, dynamic nucleocytosolic partitioning or relocalization is required for immune activation. For example, the barley ( *Hordeum vulgare* subsp. *vulgare* ) NLR protein MLA10 exhibits dynamic nucleocytoplasmic localization, with accumulation in the nucleus being essential for immune activation (Bai et al., 2012; Shen et al., 2007). Moreover, the flax ( *Linum usitatissimum* ) NLR immune receptors L6 and M localize to the Golgi apparatus and vacuolar membrane, respectively, with their N-termini determining their subcellular localization and stability (Takemoto et al., 2012). The *Arabidopsis thaliana* NLR protein RPS5 confers recognition of the protease effector AvrPphB from *Pseudomonas syringae* at the plasma membrane (Dowen et al., 2009; Qi et al., 2012). Collectively, these examples illustrate that subcellular localization often influences NLR immune output, though the relationship between localization and activation varies among NLR immune receptors.

Despite these insights, the subcellular localization of many NLR immune receptors remains unknown. Carter et al., (2019) identified a barley NLR protein termed *AvrPphB Response 1* (PBR1) that, similar to Arabidopsis RPS5, mediates recognition of the bacterial cysteine effector protease, AvrPphB. Building upon this framework, Jaiswal et al., (2023) investigated the molecular requirements for PBR1-mediated immune signaling and showed that activation of immune responses requires full-length PBR1 and intact conserved NLR motifs, thereby delineating key biochemical features necessary for PBR1-triggered immune responses. However, despite defining the structural features required for immune signaling, these studies did not address where PBR1 localizes within plant cells or whether its subcellular localization is influenced by specific domains, fluorescent tag placement, or activation state.

To address this knowledge gap, we fused the super Yellow Fluorescent Protein (sYFP) to the C-terminus of full-length PBR1 (PBR1:sYFP) and transiently expressed it in *Nicotiana benthamiana* ( *N. benthamiana* ) using *Agrobacterium tumefaciens* -mediated transformation (agroinfiltration) (Carter et al., 2019; Jaiswal et al., 2023). We previously showed that PBR1:sYFP retains the ability to activate immune signaling when co-expressed with AvrPphB (Carter et al., 2019; Jaiswal et al., 2023). Live-cell imaging using laser-scanning confocal microscopy of *N. benthamiana* epidermal cells expressing the PBR1:sYFP fusion protein consistently revealed fluorescence signals in both the nucleus and cytosol, similar to that of the free sYFP reference control ( Figure 1A- B). To determine whether individual domains or domain combinations influence subcellular localization, we expressed sYFP fusions of the coiled-coil (CC), NB-ARC, and leucine-rich repeat domains as well as the CC–NB-ARC and NB-ARC–LRR truncations. Although these fragments are incapable of activating immune signaling on their own, their localization can reveal whether loss of immune activity is associated with mislocalization. These experiments revealed that, similar to full-length PBR1:sYFP, each sYFP-tagged domain and truncation displayed nucleocytosolic distribution ( Figure 1C- G). We thus conclude that no individual domain or truncation is solely responsible for the subcellular localization of PBR1, and such localization appears to be an inherent feature of PBR1. Furthermore, our data indicates that nucleocytosolic localization of PBR1 can be uncoupled from immune activation and, on its own, is not sufficient to trigger immune signaling.

We previously showed that fusing the sYFP fluorescent epitope tag to the N-terminus of PBR1 (sYFP:PBR1) disrupts PBR1-mediated immune signaling when transiently expressed in *N. benthamiana* (Jaiswal et al., 2023). However, this work did not address whether the fluorescent epitope tag interferes with the subcellular localization of PBR1. Indeed, fusing a fluorescent tag to the N-terminus of the rice NLR protein, Pit, inhibited localization to the plasma membrane as well as compromised Pit-mediated immune signaling (Kawano et al., 2014). To investigate this, we transiently expressed sYFP:PBR1 in *N. benthamiana* and assessed its subcellular distribution using laser-scanning confocal microscopy. These experiments revealed that, similar to C-terminal tagged PBR1, the sYFP:PBR1 fusion protein also localized to the nucleus and cytosol ( Figure 1H ). We thus conclude that, though the sYFP:PBR1 protein is unable to activate immune signaling, fusing the sYFP epitope tag to the N-terminus of PBR1 does not significantly interfere with its subcellular localization.

We previously showed that mutating a conserved lysine residue within the Walker A motif of the NB-ARC domain to asparagine in full-length PBR1 (PBR1 ^K203N^ ) abolishes PBR1-mediated immune signaling when transiently expressed in *N. benthamiana* (Jaiswal et al., 2023). However, it remained possible that this mutation alters the subcellular localization of PBR1 in plant cells. Indeed, El Kasmi and colleagues (2017) showed that mutating residues within the Walker A motif altered the localization of the Arabidopsis NLR immune receptor, RPM1, from the plasma membrane to the cytosol. To rule out the possibility that suppression of immune signaling by the PBR1 ^K203N^ derivative was due to a disruption in the subcellular localization, we performed live-cell imaging of *N. benthamiana* epidermal cells expressing sYFP-tagged PBR1 ^K203N^ . These experiments revealed the PBR1 ^K203N^ :sYFP derivative consistently localized to the nucleus and cytosol, similar to that of PBR1:sYFP ( Figure 1I ). We thus conclude the K203N mutation within full-length PBR1 does not significantly affect its subcellular localization and the lack of activation of immune signaling with this derivative is not due to a disruption in the subcellular localization.

Collectively, our results demonstrate that PBR1 localizes to the nucleocytosol independently of individual domains, truncations, fluorescent tag orientation, or immune activation capacity, establishing that subcellular localization alone is not sufficient to explain PBR1-mediated immune signaling. The results presented herein provide evidence of PBR1 subcellular localization and lay the foundation for future structure-function analyses. Future work will focus on determining how PBR1 specifically recognizes AvrPphB protease activity as well as the molecular mechanisms underlying PBR1-mediated immune signaling.

## Methods

*Nicotiana benthamiana* ( *N. benthamiana* ) seeds were sown in plastic pots in Berger Seed and Propagation Mix, supplemented with Osmocote slow-release fertilizer (14-14-14). Plants were grown in a controlled growth chamber under a 16-hour light / 8-hour dark cycle with temperatures set to 24ºC and 20ºC, respectively.

The 3xHA:sYFP (free sYFP), PBR1:sYFP, CC:sYFP, NBARC:sYFP, LRR:sYFP, CC–NBARC:sYFP, NBARC–LRR:sYFP, sYFP:PBR1, and PBR1 ^K203N^ :sYFP constructs have been described previously (Carter et al., 2019; Jaiswal et al., 2023). The sequence-verified constructs were recombined into the plant expression plasmid, pBAV154, which encodes a dexamethasone-inducible promoter, and transferred into *Agrobacterium tumefaciens* GV3101 (pMP90) for transient expression assays in *N. benthamiana* (Aoyama and Chua, 1997; Jaiswal et al., 2023; Vinatzer et al., 2006).

*Agrobacterium* -mediated transient protein expression (agroinfiltration) in *N. benthamiana* was performed as previously published by Jaiswal et al., (2023) with slight modifications. Briefly, *Agrobacterium* harboring the dexamethasone-inducible constructs described above were grown on LB agar with gentamicin (25μg/mL) and kanamycin (50μg/mL) for 2 days at 30ºC. Single colonies were used to inoculate liquid LB containing the same antibiotics, and cultures were grown overnight at 30ºC with shaking at 225rpm. Cells were harvested by centrifugation, and the bacterial pellet was resuspended in 10mM MgCl _2_ , adjusted to an OD _600_ of 0.3, and incubated in 100μM acetosyringone for 3 hours at room temperature. Bacterial suspensions were infiltrated into the abaxial side of 3-week-old *N. benthamiana* leaves using a needleless syringe.

Laser-scanning confocal microscopy performed as previously published by Helm and colleagues (2022) with slight modifications. Briefly, forty hours following agroinfiltration, plants were sprayed with 50μM dexamethasone supplemented with 0.02% Tween20. Six to eight hours following dexamethasone treatment, leaf segments from *N. benthamiana* were cut and placed in sterile water between a microscope slide and coverslip, with the adaxial side oriented toward the objective. Microscopy was performed using a Zeiss LSM880 Axio Examiner upright confocal microscope equipped with a Plan Apochromat 20X/0.8 objective. The sYFP-tagged protein fusions were excited using a 514-nm argon laser and fluorescence was detected between 517-nm and 562-nm. Confocal micrographs were processed using the Zeiss Zen Blue Lite software program (Carl Zeiss Microscopy, USA).

## Reagents

**Table 1. Clones used in this study.**

**Table:** 

Name	Notes	Reference
3xHA:sYFP	Three tandem hemagglutinin epitope tag (3xHA) fused to the N-terminus of sYFP (serves as a marker for the nucleus and cytoplasm (nucleocytoplasmic)	Jaiswal et al., 2023; cloned by MH and NJ
PBR1:sYFP	Full-length PBR1 fused to the N-terminus of sYFP	Carter et al., 2019; cloned by MH
CC:sYFP x pBAV154	Coiled-coil domain from PBR1 fused to the N-terminus of sYFP	Jaiswal et al., 2023; cloned by MH and AM
NBARC:sYFP x pBAV154	NB-ARC domain from PBR1 fused to the N-terminus of sYFP	Jaiswal et al., 2023; cloned by MH and AM
LRR:sYFP x pBAV154	Leucine-rich repeat domain from PBR1 fused to the N-terminus of sYFP	Jaiswal et al., 2023; cloned by MH and AM
CC—NBARC:sYFP x pBAV154	CC—NBARC fragment from PBR1 fused to the N-terminus of sYFP	Jaiswal et al., 2023; cloned by MH and AM
NBARC—LRR:sYFP x pBAV154	NBARC—LRR fragment from PBR1 fused to the N-terminus of sYFP	Jaiswal et al., 2023; cloned by MH and AM
sYFP:PBR1 x pBAV154	Full-length PBR1 fused to the C-terminus of sYFP	Jaiswal et al., 2023; cloned by MH and NJ
PBR1 ^K203N^ :sYFP x pBAV154	Full-length PBR1 with the K203N mutation fused to the N-terminus of sYFP	Jaiswal et al., 2023; cloned by MH and NJ

**Table 2. Strain used in this study.**

**Table:** 

**Strains**	**Description**	**Source or reference**
*Agrobacterium tumefaciens* GV3101	Contains Vir plasmid encoding T-DNA transfer machinery (Rif ^r^ , Gm ^r^ )	GOLDBIO (catalog # CC-217)
